# Observations on Hypertrophy, and Other Affections of the Os Uteri

**Published:** 1839-01-19

**Authors:** 


### BIBLIOGRAPHICAL NOTICE.

*Observations on Hypertrophy, and other Affections of the Os Uteri.* By EVORY KENNEDY, M. D. Master of the Dublin Lying-in-Hospital. [Dublin Journal of Medical Science. Nov., 1838.]

The affection, to the consideration of which Dr. Kennedy's interesting paper is devoted, consists in an undue growth of the intrinsic, or fibro-cellular tissue of the lips of the os uteri, independent of any morbid change beyond the loss of balance between the secreting and absorbent vessels of the part. Contrary to the usual course of excessive uterine developement, the hypertrophy, in this case, occurs in the direction of the length of the organ, and not transversely. The aperture of the os uteri maintains its natural relations to the roof of the vagina, "the developement of the new growth projecting below the opening." The anterior lip is by far the most frequent seat of the affection, the posterior more rarely,—and "it occasionally happens that both the anterior and posterior lips become hypertrophied, and this more especially in women who have borne children. In such, the fissured or divided state of the os, that so frequently results from labour, generally continues,—the anterior and posterior lips presenting separate or distinct tumours." In one case the os uteri was "fissured into three divisions, the intermediate substance becoming hypertrophied at three points." The whole circumference of the os occasionally becomes hypertrophied in women who have not borne children; no fissure existing, such a case would naturally be confounded with prolapsus, unless a careful examination were made. The mucous membrane covering the internal parietes of the hypertrophied portion, is rugous, resembling the lining of the uterine cavity,—is often quite vascular, and frequently exhibits slight vesicles or granular growths on its surface. The outer mucous covering is polished and smooth. In one case, a peculiar appearance was presented.

"The fibro-cellular, or, as it is termed, the parenchymatous portion of the anterior lip, in its excess of developement, had enlarged into the vesico-vaginal septum, separating the bladder and vagina, in its growth, and consequently, in place of having a distinct covering of mucous membrane, it was covered by the vaginal mucous membrane at its anterior part or point only."

Hypertrophy of the os uteri may exist for some time without any peculiar symptom, although generally a particular train of symptoms is speedily manifested. A sense of fullness and weight, accompanied with burning and throbbing, aggravated at each catamenial period, are among the first indications attending this growth. As the disease progresses, the symptoms approach very nearly to those of prolapsus, and careful examination is necessary to distinguish between them. From the preternatural growth of the os uteri, its weight and bulk give to the patient the idea of a foreign body in the vaginal cavity, occasion a bearing down sensation, and are productive of much irritation. When the anterior lip is involved, the functions of the bladder become impeded, and micturition and retention of urine occur. When the posterior lip is the seat of disease, costiveness, or pain at stool, are the consequences. Here retroversion or anteversion of the uterus are simulated. The menstrual discharge usually continues regular, and, occasionally, menorrhagia occurs. Leucorrhœa is almost a constant complication, and much pain is experienced in coition. On examination with the speculum, a simple increase of bulk is the only appreciable appearance in some cases; in others we find a highly vascular condition of the hypertrophied part, with granular disease.

Hypertrophy of the os uteri is very likely to be mistaken for polypus of that organ, from the great extent of its unattached surface, and from its connexion with the womb being at its superior portion. It may be distinguished from intra-uterine polypus, by its external connexion with the womb, and from its attachment not being pediculated, it differs from polypus of the neck or os tincæ. To the amount of sensibility present Dr. Kennedy does not attach any importance in diagnosticating between the two diseases, as its absence is but an equivocal test for polypus, and great insensibility is frequently manifested in hypertrophy.

In the treatment of this affection, where there is any thing approaching to a congestive or inflammatory condition, local depletion, warm hip-baths, saline aperients, a protracted use of alteratives, anodynes, and a strict adherence to the horizontal position, must be employed. Dr. Kennedy has found washing the hypertrophied part with a strong solution of lunar caustic, or the application of the solid caustic to it, to be of service. Should these means fail, and serious symptoms occur, then the removal of the hypertrophied portion must be had recourse to. To guard against excessive hæmorrhagy, Dr. Kennedy applies first a ligature, and removes the tumour below the strictured part with a pair of curved scissors, the blades of which are placed at an angle with their stalks.

In the impregnated female, hypertrophy of the os uteri is very frequently met with, and deserves to be ranked among the complications of labour. Protruding before the head of the child into the vagina, it is often mistaken for an extraordinary presentation, placenta, or polypus. Its texture, in such cases, is loose and soft, and during parturition, especially when prolonged, communicates the sensation of fluctuation, giving the idea of a tumour containing fluid.—As in the unimpregnated state, the anterior lip is the usual seat of the affection. The degree of thickness varies from three lines to nearly an inch, and it is from half an inch to four inches in length. It is commonly broad at its point of connection with the uterus, yet sometimes its base is narrow, and resembles very much a polypous growth.

Where the hypertrophied os uteri arrests the progress of the labour, Dr. K. has found a very simple manipulation to answer—to press the protruding lip gently upwards, and retain it with the fingers, during two or three pains. If this should fail, "a small piece of sponge tint may be pressed up, so as to rest at the upper part of the space through which the lip protrudes, and prevent its relapse." Where it is impracticable to accomplish this from the extent of the protrusion, or from want of space between the head and pubis, he advises puncturing, with a lancet, the protruded lip. Sometimes edema of the lips of the os uteri occur, with astonishing rapidity, during the progress of a difficult labour. Here a few punctures will evacuate the fluid, and hasten, materially, labour.

It is not, sometimes, until after delivery, tha these conditions are discovered by the practitioner, and then "a deeply colored fleshy mass" is found within the vagina, or beyond the vulva. This is mistaken for a prolapsus, or inversion of the womb, or for polypus.

"It is to be diagnosed by introducing the fingers, or hand, if necessary, into the vagina, when having ascertained that the uterus is in its natural position, by feeling the os and neck, we must satisfy ourselves that the connection of the protruding tumour is with the neck of the uterus by an extended base, not a pedicle, as in polypus. In fact, the examinator must satisfy himself of its being a continuation or elongation of the lips downwards, by an excess of growth. The production is generally less tumid and thick, or rather more flaccid, just after delivery than during labor."

In these cases, nature is usually competent to effect a cure herself; sufficient time should therefore be allowed to elapse after parturition, before the practitioner attempts to interfere, "and even then, if it continue, he should not unnecessarily remove the hypertrophied portion, unless it do not yield to the treatment before recommended, or inconvenience shall occur from it to a sufficient extent to justify the operation." The longer the delay the less risk is run, for, from the contraction of the womb, the diminished bulk of the tumour will render the operation easier.

We hope to have other opportunities of presenting to our readers the result of the observations of so practical and accomplished an obstetrician as Dr. Kennedy.

